# Vision-force-fused curriculum learning for robotic contact-rich assembly tasks

**DOI:** 10.3389/fnbot.2023.1280773

**Published:** 2023-10-06

**Authors:** Piaopiao Jin, Yinjie Lin, Yaoxian Song, Tiefeng Li, Wei Yang

**Affiliations:** ^1^Department of Engineering Mechanics, Center for X-Mechanics, Zhejiang University, Hangzhou, China; ^2^Hikvision Digital Technology Company, Ltd., Hangzhou, Zhejiang, China

**Keywords:** contact-rich manipulation, multimodal perception, sensor fusion, curriculum learning, robotic assembly task

## Abstract

Contact-rich robotic manipulation tasks such as assembly are widely studied due to their close relevance with social and manufacturing industries. Although the task is highly related to vision and force, current methods lack a unified mechanism to effectively fuse the two sensors. We consider coordinating multimodality from perception to control and propose a vision-force curriculum policy learning scheme to effectively fuse the features and generate policy. Experiments in simulations indicate the priorities of our method, which could insert pegs with 0.1 mm clearance. Furthermore, the system is generalizable to various initial configurations and unseen shapes, and it can be robustly transferred from simulation to reality without fine-tuning, showing the effectiveness and generalization of our proposed method. The experiment videos and code will be available at https://sites.google.com/view/vf-assembly.

## 1. Introduction

In recent years, there has been a growing interest in developing advanced robotic systems capable of performing complex assembly tasks (Sergey et al., 2015; Oikawa et al., 2021; Spector and Zacksenhouse, 2021). These tasks often involve intricate manipulation of objects in contact-rich environments, requiring the robot to possess a high degree of dexterity and adaptability. The success of contact-rich assembly tasks relies on a combination of accurate perception, precise control, and intelligent decision-making. Robots must be equipped with sensory capabilities that enable them to perceive and understand their environment, such as vision systems that capture high-resolution images or depth maps (Morrison et al., 2019; Andrychowicz et al., 2020; Zeng et al., 2021). Additionally, force perception and control mechanisms play a crucial role in managing the physical interaction between the robot and the objects, ensuring gentle and accurate manipulation (Raibert and Craig, 1981; Whitney et al., 1982; Hogan, 1984; Khatib, 1987).

While significant progress has been made in the utilization of unimodal approaches, focusing solely on vision or force (Chhatpar and Branicky, 2001; Tang et al., 2016; Bogunowicz et al., 2020; Stevŝić et al., 2020; Xie et al., 2023), the integration of these modalities presents a compelling opportunity for robots to exploit the complementary nature of vision and force information. By integrating these modalities, robots can enhance their perception and control capabilities, enabling them to adapt effectively to uncertain and dynamic environments. There are two primary approaches to integrating these two modalities: sensor-based controller integration and sensory data fusion (Hosoda et al., 1996). Firstly, visual servoing control and force control are designed separately to form a result scheme capable of coordinating two sensors, and a hybrid structure of sensor-based controllers is built accordingly. Gao and Tedrake (2021) extract the key point representation of the object with a visual detector and then command the robot to the desired pose with the force controller. However, this decoupling method of pose control and force perception ignores the fact that the contact force aroused during the interaction helps to localize the target pose and may enhance the performance of the control scheme. Secondly, given the prioritization of external sensor-based controller coordination over sensory data coordination during the perception phase (Hosoda et al., 1996), this kind of method remains underdeveloped until the emergence of data-driven methodology. This methodology facilitates the fusion of modalities, irrespective of their individual characteristics, and has sparked a surge of interest in numerous studies focusing on robotics perception (Van Hoof et al., 2016; Lee et al., 2020a; Song et al., 2021; Zhao et al., 2021; Spector et al., 2022).

To overcome the limitations of the aforementioned existing methods, we consider a holistic approach to unifying the perception and control modeling process for contact-rich assembly tasks. Specifically, a novel robotic framework based on multimodal fusion and curriculum learning is proposed to improve the performance of contact-rich policy generation end-to-end. Firstly, multimodal perception (i.e., vision and force) are considered to extract multimodal fusion features. Next, we employ reinforcement learning techniques (Sutton and Barto, 2018) to generate both motion and force commands reactive to the multimodal features. For efficient multimodal policy learning, our method includes a two-step vision-force curriculum learning (CL) scheme (Bengio et al., 2009), allowing agents to learn from a curriculum of tasks that progress in complexity and difficulty. The acquired policy is then implemented by a Cartesian motion/force controller, an innovation from our prior work (Lin et al., 2022), designed to guarantee compliant movements amidst uncertain contacts.

To acquire the multimodal policy, we propose a simulated assembly environment based on MuJoCo (Todorov et al., 2012), where the multimodal fusion and policy generation mechanisms are developed. After learning the multimodal policy in simulation, we transfer the simulated system to its physical counterpart. Our multimodal perception-control system could handle the imperfect modeling of interactions in simulated contact-rich scenarios and demonstrate the possibility of a direct sim-to-real transition using a variety of domain randomization techniques (Peng et al., 2018; Chebotar et al., 2019). To evaluate the effectiveness of our proposed framework, a comprehensive series of experiments are conducted on both simulated and physical robots. The results illustrate the remarkable capabilities of the vision-force perception and control system in the simulated environment. It achieves an impressive success rate of 95.3% on a challenging square assembly task whose clearance is 0.1 mm. Furthermore, the algorithm exhibits robust generalization across various spaces, sizes, and even previously unseen shapes. Most notably, the simulated system is seamlessly transferred to the physical environment, achieving zero-shot capabilities and highlighting its potential for real-world implementation.

In summary, the contribution of this work could be summarized as below:

- We propose a novel vision-force framework for contact-rich assembly tasks, enabling multimodal perception and control in challenging and precise operations.
- We introduce a vision-force-fused curriculum learning approach, which progressively coordinates multimodal features based on task difficulty. This innovative approach enables effective vision-force fusion and policy learning specifically tailored to precise assembly tasks.
- We conduct extensive experiments to validate the efficacy of our proposed method. The vision-force perception and control system demonstrates robust generalization capabilities across varying poses and previously unseen shapes. Moreover, we successfully transfer the control scheme to real-world scenarios, ensuring its reliability and applicability in practical settings.

## 2. Related work

### 2.1. Force and vision perception in the assembly task

For unimodal perception and control, several methods develop force controllers and map the contact force to misalignment between the peg and the hole (Tang et al., 2016; Inoue et al., 2017). Unten et al. (2023) accurately estimate the relative position between the peg and hole through the force/torque sensing from the transient responses. However, the above methods require prior knowledge of geometry and fail to generalize over new shapes. Apart from the use of force, the utilization of vision to search for holes has also been investigated (Schoettler et al., 2019; Nair et al., 2023). Utilizing an in-hand RGB-D camera, Zhang et al. (2023) develop a 6-DoF robotic assembly system for multiple pegs.

For multimodal perception and control, the complementary nature of vision and force inspires a flurry of study on how to utilize better visual and force sensory feedback. The normal practice is to control the force along the constraint direction while controlling motion via visual servoing along the remaining directions (Haugaard et al., 2021). The task geometry needs to be known a priori in order to properly design the controller through a selection matrix that ensures orthogonality between vision and force control directions. The combination of visual servoing control and impedance control is also actively proposed. The position of the hole is estimated using two depth cameras, followed by a spiral search for the hole using impedance control in Triyonoputro et al. (2019). However, the aforementioned algorithms only combine disparate sensors with their respective controllers. This sensory data separation does not fully exploit the complementarity of vision and force. To better coordinate vision and force, several works have focused on combining visual servoing control and force regulation to achieve a fusion of visual and force perception. The External/Hybrid vision-force control scheme is developed to reach visual and force references simultaneously (Mezouar et al., 2007). The external wrench is transformed into a displacement of the image's feature reference. And all directions of the task space are simultaneously controlled by both vision and force. Oliva et al. (2021) further generalize the control scheme by not specifying the visual features.

This paper takes a different approach by simultaneously leveraging visual and force features to generate compliant motion and force commands. The system's capability to accommodate environmental variations is greatly expanded as the accurate interaction model is unnecessary in our approach.

### 2.2. Reinforcement learning-based manipulation

Reinforcement learning (RL) endows robots the promise to accommodate variations in environmental configurations. Some previous works on impedance, admittance, and force control are revisited under the RL scope (Luo et al., 2019; Zang et al., 2023). Oikawa et al. (2021) extend the traditional impedance control using a non-diagonal stiffness matrix learned over RL for precise assembly. Similarly, the use of RL in the admittance control trains the deep neural network that maps task specifications to corresponding parameters (Spector and Zacksenhouse, 2021). Although these algorithms could handle uncertainty and achieve the task, the validness of the unimodal methods is restricted to the single modality's functioning ranges. The development of multimodal policy holds the potential to further enhance manipulation ability (Luo et al., 2021). Lee et al. (2020b) learn a representation model that combines vision, haptics, and proprioceptive data. The state representation is validated in peg-in-hole insertion tasks. Nevertheless, the complicated multimodal features and tedious fine-tuning may hinder practical applications. To simplify the multimodal policy learning process, some strategies leverage prior task knowledge or human demonstrations (Zhao et al., 2021; Spector et al., 2022). Despite their impressive performance in physical insertion experiments, these approaches necessitate human interventions, which are infeasible to acquire in hazardous environments.

Despite the potential of acquiring general policies with RL, the sample inefficiency of RL results in tedious policy training and ill-posed real machine deployment. To overcome the disadvantage, model-based methods (Luo et al., 2019) have been utilized by several researchers to fill this gap, avoiding extensive interactions and training. Curriculum learning (CL) which allows the agents to learn from a curriculum of tasks that progressively increase in complexity and difficulty, could facilitate learning efficiency and improve manipulation performance. Dong et al. (2021) train the insertion agent in progressively more complex environments (wall → corner → U → hole). The result shows that the curriculum training scheme improves the data efficiency of RL and made the problem feasible to solve in a reasonable training time.

In this paper, we propose a novel framework for multimodal curriculum policy learning which could not only explore the compatibility of vision and force but also achieve effective multimodal decision-making. The method is free of human interventions and task priors that expand the scheme's applicability. To effectively deploy the method on the real machine, we train the system in the simulation and then transfer the trained policy to reality. The inconsistencies in perception and control in simulated and real environments (called the reality gap) are bridged by domain randomization (Peng et al., 2018).

## 3. Problem statement

**Figure 1:**
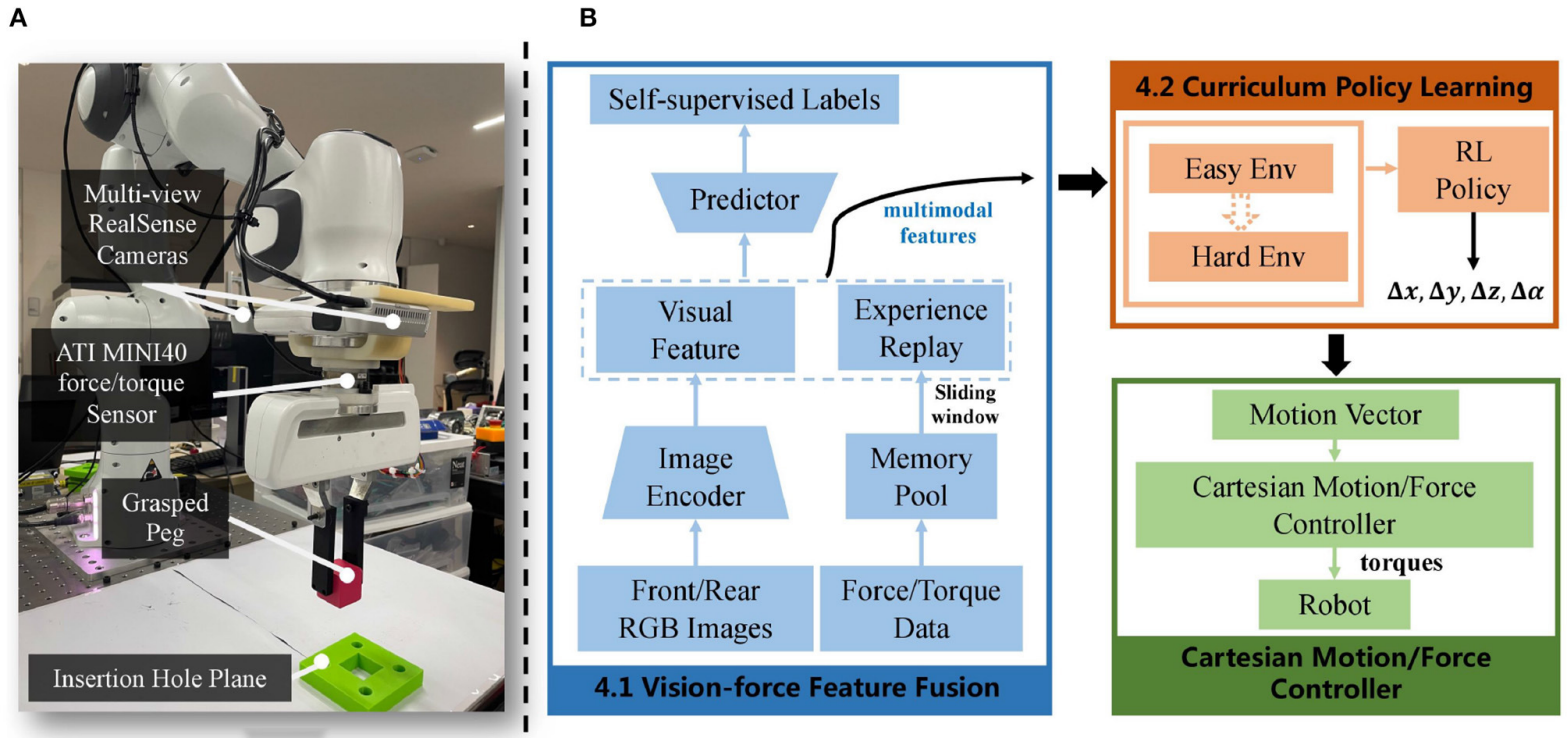
**(A)** Setup of the task: the experimental setup comprises a Franka Emika Panda robot arm equipped with two wrist-mounted RealSense D435 cameras for vision perception and a six-axis ATI mini40 force/torque sensor for interaction forces capturing. **(B)** The overview of our framework includes vision-force feature fusion (blue), followed by curriculum learning-based policy generation (orange), and ended with the motion vector execution module using a Cartesian motion/force controller (green).

Our algorithm aims to develop a vision-force perception and control system and validate the scheme in the assembly task. The task is to insert the grasped square peg into the corresponding hole whose clearance is up to 0.1 mm and depth up to 10 mm as shown in Figure 1. Starting from a randomized robot arm configuration, the robot must maneuver and rotate the peg to insert into the target hole, which could be denoted as *robot*_*init*_ → *hole*_*target*_. To reach *hole*_*target*_, we formulate the task as a servoing problem and generate the incremental motion vector Δ*X* at each timestep. The desired robot pose *X*_*target*_ could be derived from the current robot pose *X*_*cur*_ as:


(1) $$\begin{array}{l} X_{target}=X_{cur}+\text{Δ}X,\\ \text{ Δ}X=f\left(x_{v},x_{f}\right), \end{array}$$

where *x*_*v*_ and *x*_*f*_ represent raw vision and force observation from robotic sensors, respectively. *f* is the function mapping from the raw sensory data to the motion vector Δ*X* ∈ R^4^ (i.e. [Δ*x*, Δ*y*, Δ*z*, Δθ]), where Δ*x* represents the incremental displacement along *x*-axis, and so does Δ*y* and Δ*z*. Δθ represents incremental *z*-axis roll command. Absent any prior information about the hole's geometry and pose, the robot must rely solely on sensory feedback to generate motion vector Δ*X*. Since the robot exhibits distinct dynamic properties before and during contact, some methods split the task into two stages: vision-based hole searching in the free space and force-based insertion in the constraint space. In contrast, our method proposes a single strategy that unifies the two stages, eliminating the need for prior knowledge of how to solve the task and simplifying the modeling process.

Nevertheless, unifying the two stages and devising a single policy function *f* is quite challenging because visual and force data exhibit different characteristics in the two stages. Therefore, this paper explores the utilization of modality-specific encoders to fuse vision and force and curriculum policy learning to generate motion commands progressively. By leveraging modality-specific encoders, visual and force features are extracted from *x*_*v*_ and *x*_*f*_, respectively. Through curriculum policy learning, the policy function π_*mlp*_ automatically generates motion vector Δ*X* based on the concatenation of visual and force features as shown in Equation (2).


(2) $$\begin{array}{l} \phi_{v}=E_{vision}\left(x_{v}\right),\\ \phi_{f}=E_{force}\left(x_{f}\right),\\ \text{Δ}X=\pi_{mlp}\left(\phi_{v}⊕\phi_{f}\right), \end{array}$$

where *E*_*vision*_ and *E*_*force*_ represent the visual and force encoders, respectively. *ϕ*_*v*_ and *ϕ*_*f*_ the extracted visual and force features, while (*ϕ*_*v*_⊕*ϕ*_*f*_) concatenation of visual and force features. To this end, the initial servoing problem defined in Equation (1) is transformed into investigating modality-specific encoders and a vision-force-fused curriculum policy learning scheme to generate the incremental motion vector. As such, the target motion vector is derived as in Equation (3). The target motion vector *X*_*target*_ is then executed by the Cartesian motion/force controller proposed in our previous work (Lin et al., 2022).


(3) $$\begin{array}{l} X_{target}=X_{cur}+\pi_{mlp}\left(\phi_{v}⊕\phi_{f}\right). \end{array}$$

## 4. Method

As is shown in our control framework Figure 1, our method begins by using modality-specific encoders to extract visual and force features. These features are then combined to form the multimodal features (Section 4.1). Next, the curriculum policy learning mechanism is employed to train an assembly policy, which hierarchically uses the multimodal features in an environment that gradually increases in difficulty (Section 4.2). Lastly, to execute the motion vector, we utilize the Cartesian motion/force controller proposed in our previous work (Lin et al., 2022). The implementation details are explained in Section 4.3. By coordinating vision and force in the generation and execution of the motions, our vision-force perception and control scheme could fully utilize the multimodality and form a resultant robust assembly system.

### 4.1. Vision-force feature fusion

The heterogeneous nature of visual and force sensory feedback requires modality-specific encoders to capture the unique characteristics of each modality. We design modality-specific encoders and fusion modules to approximate Equation (2). For the force encoder *E*_*force*_, we employ experience replay with a sliding window of the most recent five frames to extract the force feature. The aggregated force signals are later flattened to a 30-dimensional force feature *ϕ*_*f*_. Compared to the instant F/T data, the experienced force/torque (F/T) sensory data within the time windows provides a more compact representation of the robot-environment interactions. To further process the data, the raw force data is normalized with the mean (*f*_μ_) and variance ($f_{\sigma^{2}}$). The tanh function further scales the data between −1 and 1.

**Figure 2:**
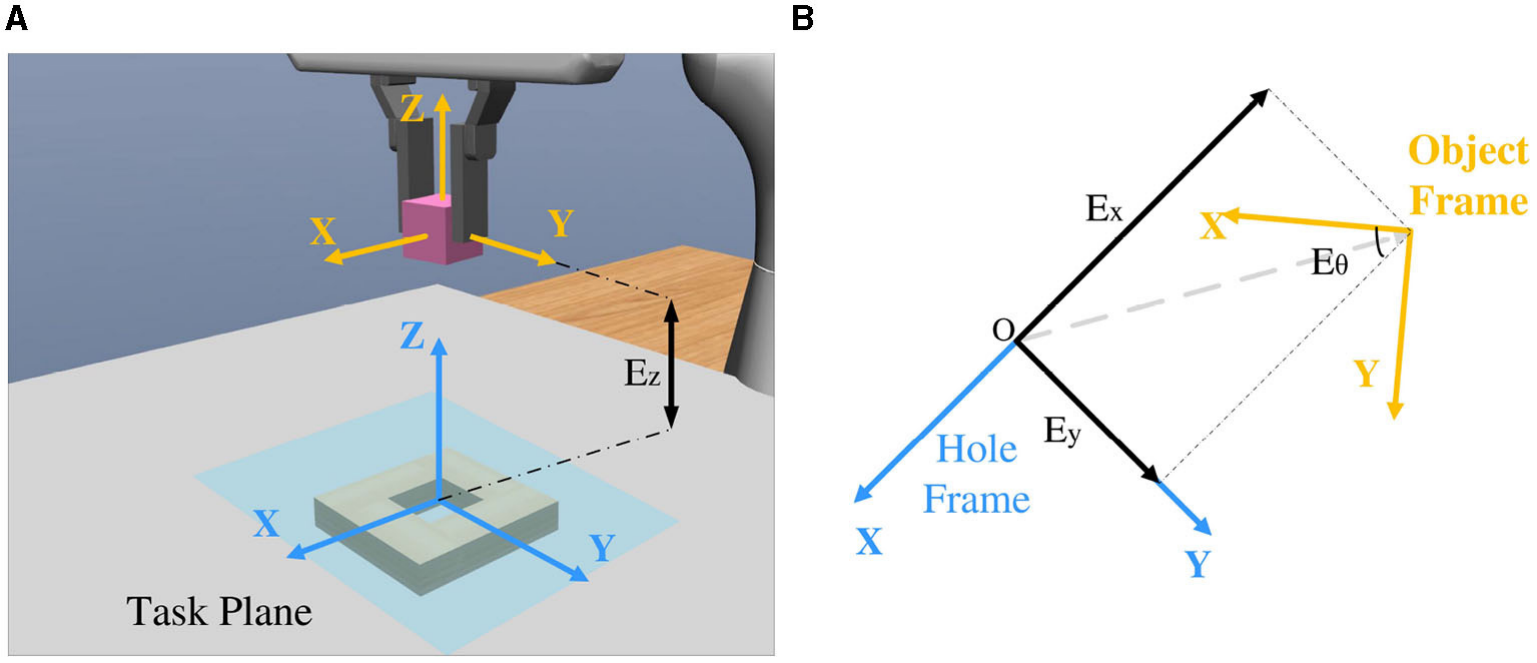
**(A)** Frames of the hole and object in the simulator MuJoCo. **(B)** The transformation between the hole and object frames is denoted by four parameters, *E*_*x*_, *E*_*y*_, *E*_*z*_, and *E*_θ_.

For the visual encoder *E*_*vision*_, we propose a self-supervised algorithm to extract its RGB feature *ϕ*_*v*_. As shown in Figure 1, two cameras are symmetrically placed to the gripper. From the top-down view, the grasped peg and hole are observable from the images. With these two images, the visual feature related to the spatial relationship between the peg and hole can be extracted. The spatial relationship between the grasped peg and hole could be denoted by four parameters, *E*_*x*_, *E*_*y*_, *E*_*z*_, and *E*_θ_, which individually represent the translation error along the *x*, *y*, and *z* axes, as well as the *z*-axis rotational error (Figure 2). To extract the visual feature, the self-supervised neural network predicts three Booleans related to *E*_*x*_, *E*_*y*_, and *E*_θ_, while *E*_*z*_ is not observable due to the loss of depth information. Rather than regressing to the values of *E*_*x*_, *E*_*y*_, and *E*_θ_, the outputs indicate whether they are positive or negative. More precisely, a label of 0 is assigned when the value is negative, and a label of 1 is assigned when the value is positive.

**Figure 3:**
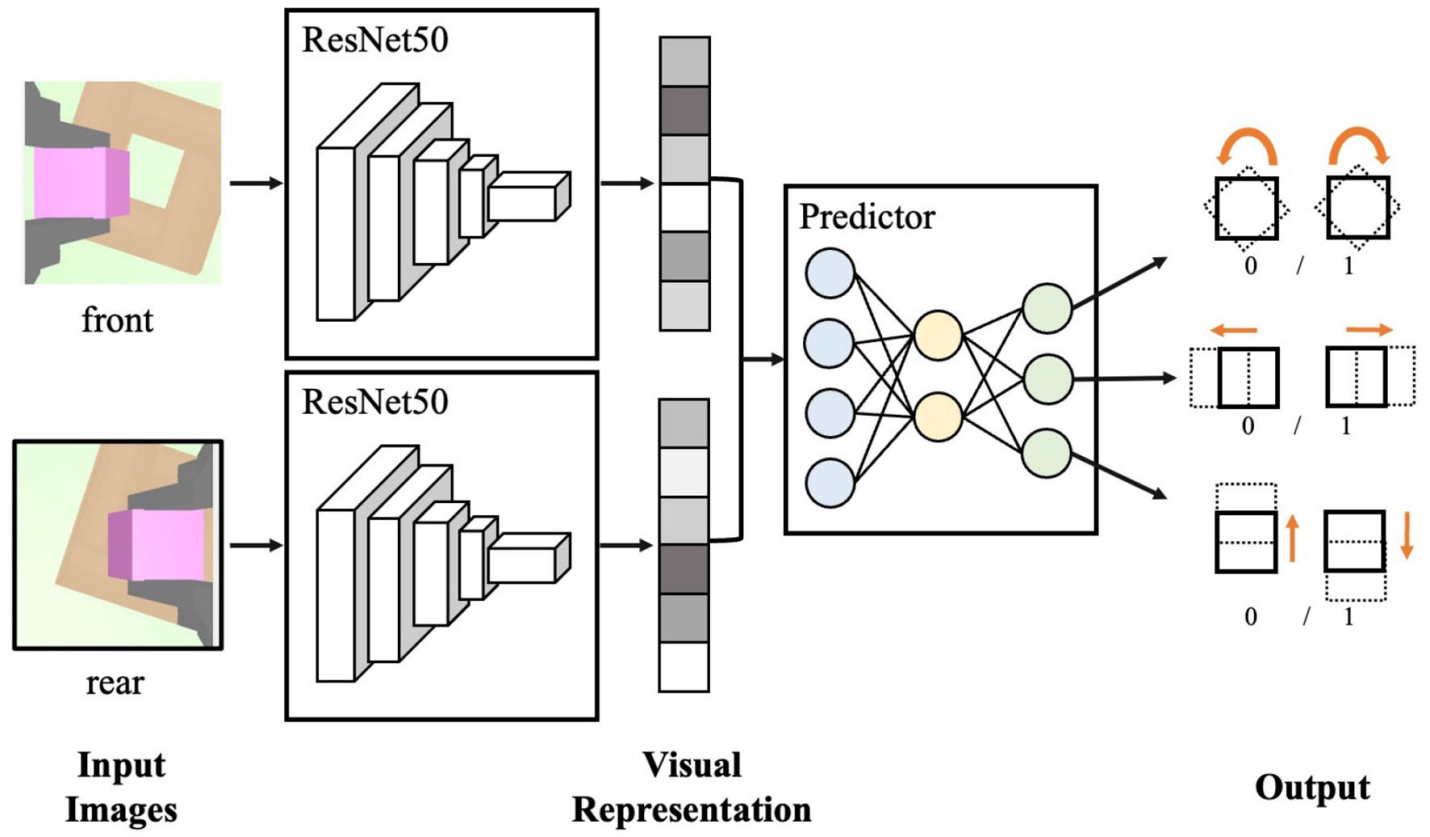
The neural network architecture of the self-supervised visual encoder.

As illustrated in Figure 3, the first step is to crop two RGB images to a size of 224 × 224. These images are then processed individually using the ResNet50 backbone network (He et al., 2016) and reduced to a 128-dimensional feature space. The resulting visual feature is subsequently input to a three-layer multi-layer-perceptron (MLP) to predict the spatial relationship between the grasped peg and the hole. To train the self-supervised visual neural network, the dataset comprising 60k synthetic multi-view RGB images and labels is collected in the simulation. While this simplifies the labor of performing the operation on real machines, the reality gap of the images hinders the direct transfer of the synthetic visual system to the real robot. To bridge the reality gap, a series of domain randomization techniques are applied, such as Gaussian blurring, white noise, random shadows, and random crops. What's more, in simulation, the colors of the peg, hole, and background are also randomly varied.

### 4.2. Curriculum policy learning

Our goal is to enable robots to perform precise assembly tasks leveraging visual and force sensory feedback. To achieve the goal, we utilize deep reinforcement learning to map the visual and force sensory data to the robot's motion vector and guide the robot to the target pose following Equation (3). The input to the multimodal policy is the fusion of the visual and force features (*ϕ*_*v*_⊕*ϕ*_*f*_) as defined in Equation (2). π_*mlp*_ is the multi-layer-perceptron (MLP) function mapping the sensory features to the incremental robot vector Δ*X*. To learn the policy, the assembly task is formulated as a model-free reinforcement learning problem. This approach avoids the need for an accurate dynamics model that is typically hard to obtain due to the presence of rich contacts. Furthermore, we apply curriculum learning (CL) to structure the task difficulty in accordance with the sensory data input so as to facilitate learning efficiency and enhance model performance. The algorithm is detailed in Algorithm 1.

**Algorithm 1:** Vision-force-fused curriculum policy learning.

** 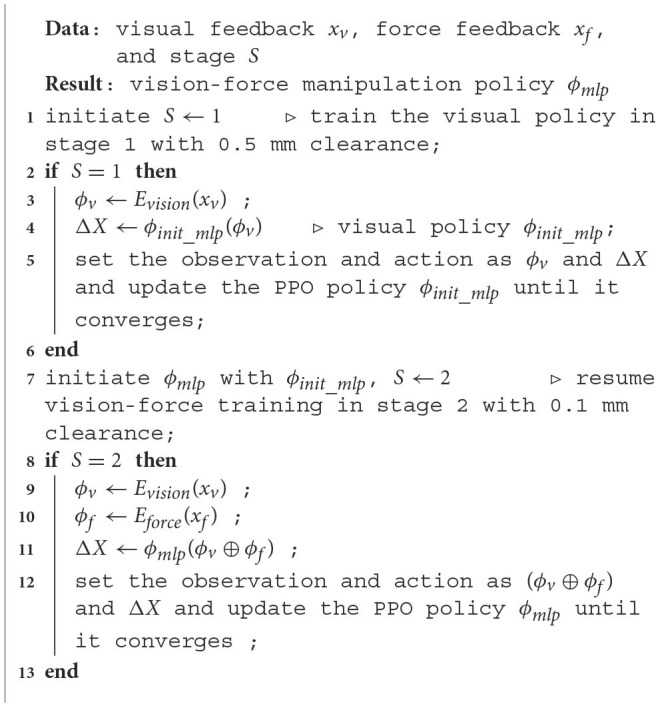 **

**Figure 4:**
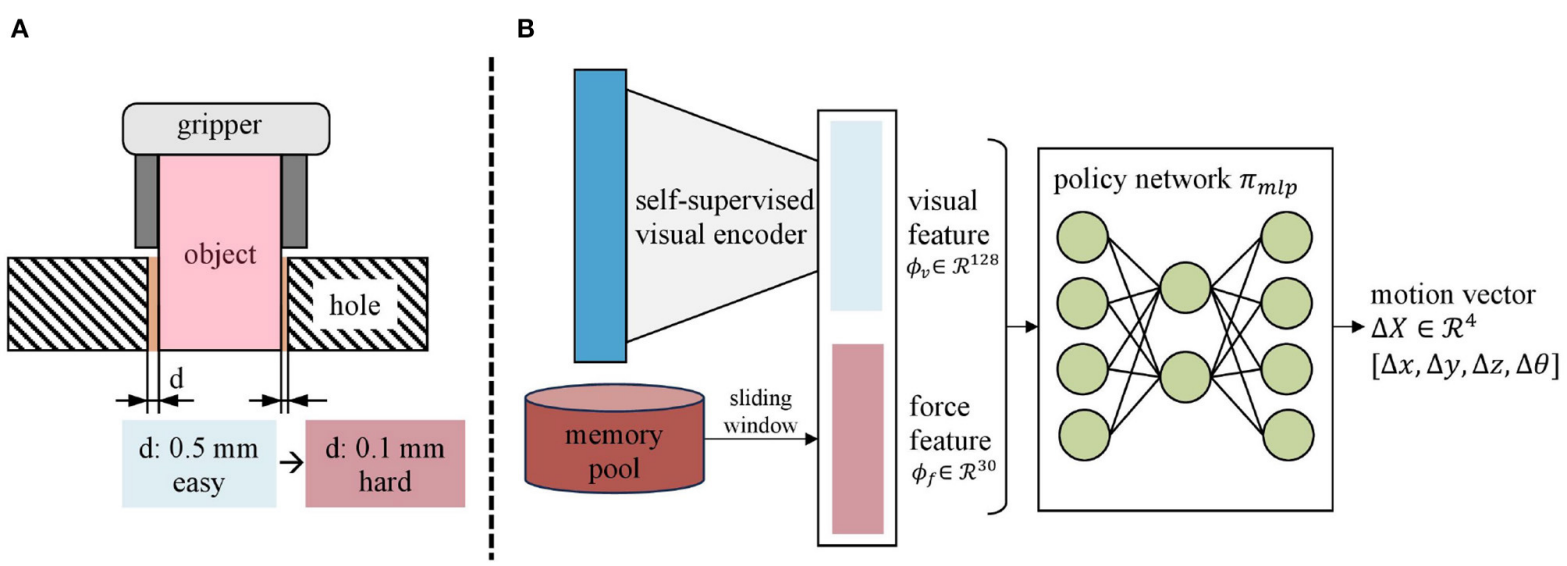
The curriculum policy learning procedure. **(A)** The clearance influences policy learning critically. **(B)** Firstly, the peg-hole clearance *d* is 0.5 mm and the observation is a 128-dimensional visual feature *ϕ*_*v*_. Secondly, the peg-hole clearance is narrowed to 0.1 mm and the observation space is expanded with the incorporation of force feature *ϕ*_*f*_. The action space is a four-dimensional motion vector Δ*X* consisting of the desired displacement along the *x*, *y*, and *z* axes and the *z*-axis rotation roll.

The CL approach divides the training process into two stages: the pure visual policy learning stage and the continued vision-force policy learning stage (shown in Figure 4 and Algorithm 1). The observation space of the first stage contains only 128-dimensional visual feature *ϕ*_*v*_ (Section 4.1), and the larger peg-hole clearance makes this stage of the task easier to manipulate. The difficulty of the second stage intensifies by narrowing the peg-hole clearance to 0.1 mm. We extend the observation space to 158 dimensions by combining the 30-dimensional force feature *ϕ*_*f*_ (Section 4.1). The visual strategy learned in the first stage provides a rough translational and rotational relationship between the grasped peg and the hole. After mastering the required skills in the first stage, the robot proceeds to train in more challenging scenarios incorporating force data. The training in the second stage is like fine-tuning the global visual policy with the local contact force. The action space Δ*X* for both stages is a 4-dimensional vector representing the desired displacements along *x*, *y*, and *z* axes, and the *z*-axis rotation roll in the object frame (Δ*X* = [Δ*x*, Δ*y*, Δ*z*, Δθ]). Meanwhile, to achieve compliance along the *z*-axis, we command the interaction force along the *z*-axis to be zero. The Cartesian motion/force controller proposed in Lin et al. (2022) executes the motion and force commands.

Although complex reward functions are often devised for reinforcement learning algorithm (Lee et al., 2020b), sparse rewards are sufficient in our proposed method experimentally. Specifically, the agent obtains the reward of 0.5 if the peg is aligned with the hole and half inserted. The agent gets another reward of 0.5 if the peg is entirely in the hole. Besides, if the peg falls off the gripper, the agent will receive a penalty of −0.2. Since in our setup, the peg is grasped and not fixed to the gripper. The peg can easily fall off the gripper if a large contact force and undesired movements occur.

### 4.3. Implementation details

To train the self-supervised visual encoder *E*_*vision*_ proposed in Section 4.1, we use a binary-class cross-entropy loss to optimize the network with Adam optimizer. We train the network for 20 epochs with batch size 32 and learning rate 1*e*^−4^ under PyTorch 1.11. To achieve a more generalized and robust policy π_*mlp*_ (Section 4.2), simulation training is conducted under diverse conditions. The initial relative pose of the peg and hole is sampled from a uniform distribution. Specifically, the pose error along the *x* and *y* axes is randomly distributed between −10 mm and +10 mm, while the *z*-axis positional error is distributed between 5 mm and 20 mm. The *z*-axis rotational error is uniformly distributed between −10° and +10°. It is assumed that the gripper has already grasped the peg using a human-designed grasp pose. To introduce additional positional randomness, errors along the *x* and *z* axes are uniformly distributed between −2 and +2 mm. The training of the policy employs Proximal Policy Optimization (PPO) (Schulman et al., 2017), implemented using the stable baselines library (Hill et al., 2018). In training the PPO algorithm, the n_steps is chosen to be 64, and the batch_size is 32, and the gae_lambda to be 0.998.

## 5. Experiment

We conduct simulated and physical experiments to evaluate the performance and effectiveness of our vision-force perception and control system for the contact-rich assembly task. In particular, we investigate the following four research questions (**RQs**):

- RQ1. How does our proposed method outperform existing work in contact-rich assembly tasks?
- RQ2. Is the multimodal-based policy robust to unseen shapes, colors, and places?
- RQ3. How do modules of our proposed framework improve the final performance?
- RQ4. Can our proposed method perform well in real-world scenarios?

### 5.1. Evaluation metrics

We define a trial as successful if the robot effectively navigates the peg, securing it within the hole to a depth of 10 mm. Conversely, a trial is considered unsuccessful if the peg slips from the robot's grasp, preventing its insertion into the hole.

### 5.2. Simulation results analysis

For **RQ1**, we initially evaluate the performance of our vision-force system in the square peg insertion task and then compare the results with those of existing vision-force assembly systems, enabling a comprehensive assessment of the proposed approach. Experimental results indicate that our proposed method outperforms existing baseline work broadly. As shown in Table 1, comparing our method with the baseline from Lee et al. (2020b), we achieve more than 15% improvement in *success rate* (78% → 95.2%). Their method is consistent with ours in fusion vision and force perception and adoption of an impedance controller for incremental motion execution. Nevertheless, they utilize naive RL for policy training while we take a CL approach and split the task into two parts to learn the insertion strategy progressively. Moreover, our Cartesian motion/force controller is more advantageous when dealing with unknown contacts. These two major aspects explain our model's great outperformance. For *clearance*, our method improves 50% relative to baseline from Gao and Tedrake (2021) (0.2 mm → 0.1 mm). Their approach involves a vision-based key point detector followed by a force controller. Our approach differs in formulating the insertion task as a servoing problem and making decisions leveraging both visual and force data end-to-end, thereby achieving more precise manipulation. Although our approach doesn't achieve the high success rate as the work in Spector et al. (2022), our method doesn't require human demonstrations and prior task information. Moreover, our evaluation metrics are stricter by requiring a 10 mm insertion depth while the work in Spector et al. (2022) only requires a 1 mm insertion depth.

**Table 1:** The performance of different multimodal models in the assembly task.

**Models**	**Clearance ↓**	**Peg**	**Modalities**	**DoF**	**Success rate ↑**	**Shape generalization**	**Human demonstration**
Gao and Tedrake (2021)	0.2 mm	Unfixed	RGB/depth/force	3	74%	No	No
Lee et al. (2020b)	2 mm	Fixed	RGB/depth/force	4	78%	Yes	No
Spector et al. (2022)	–	Unfixed	RGB/force	6	**97.5** **%**	No	Yes
Ours	**0.1 mm**	Unfixed	RGB/force	4	95.2%	Yes	No

The bold values represent the best performance among the comparisons.

**Figure 5:**
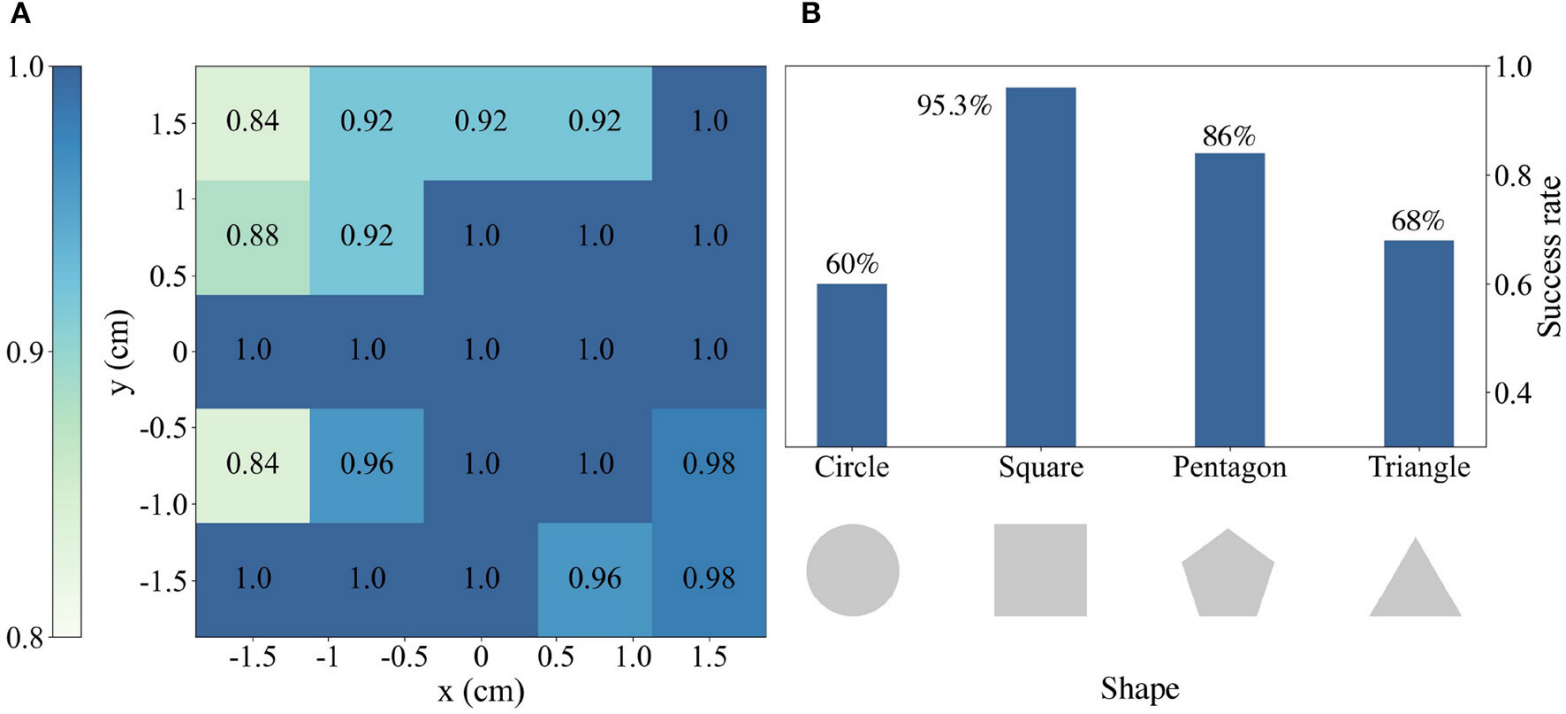
**(A)** Simulation experimental results with varied initial positions for peg-hole operations using a square object. Each individual value corresponds to the insertion success rate at that region, thereby providing a comprehensive overview of the spatial distribution and variations in success rates of the square peg insertion task. **(B)** The success rate of different peg-hole objects, in which square is used in training (in-domain) while others only are used to test (out-of-domain).

For **RQ2**, we first conduct a series of insertion tasks initiating with a randomized peg-hole position error within [−15 mm, 15 mm] along both *x* and *y* axes. At each position, we conduct 50 trials to statistically evaluate the system's performance. Next, we test the system's out-of-domain performance on three different shapes that have never been exposed before, namely the pentagonal, triangular, and circular pegs. Experimental results demonstrate that our multimodal system is robust to varying in-domain initial configurations and novel shapes. As shown in Figure 5A, our method achieves an overall success rate of 95.2% across the varying initial pose errors up to 3 cm, which is a reasonable setup in factories and social industries. When the positional error is small than 1.5 cm, the success rate even reaches nearly 100%. The method's robustness to varying positions owns the object-centric design of the observation and action. Specifically, the observation and action are centered on the object coordinate regardless of the robot configurations and global positions. As long as the hole plane can be observable from the in-hand cameras, the robot is able to approach the hole. For novel shapes, the result in Figure 5B indicates the method's remarkable robustness to unseen shapes. Although the novel shapes are never explored before, they share similar task structures with the square pegs. Among the three new shapes, the pentagonal peg is most similar to the square peg and thus has better generalization ability than the other shapes. The triangular peg insertion task is more challenging with a higher *z*-axis roll requirement. Surprisingly, the model behaves poorly on the circular peg, probably due to the small contact surface (line contact) between the peg and the gripper. Although the hardware setup for the circular peg easily causes slippage and tilt, it still maintains a success rate of 60%.

### 5.3. Ablation study of proposed module

For **RQ3**, we investigate the contributions of the design choices, namely the act of vision-force perception fusion and the curriculum vision-force fusion mechanism. This section conducts two comparisons: (1) we compare whether the fusion of vision and force boost performance over vision only. (2) we investigate whether the two-stage curriculum learning (CL) fusion mechanism could improve fusion efficiency and manipulation performance than the naive reinforcement learning (RL) fusion mechanism. To verify the suppositions mentioned above, we design the following models:

- **Vision-only CL model** contains only vision perceptually and curriculum learns the visual policy.
- **Vision-force CL model** curriculum learns the vision-force multimodal policy.
- **Naive RL model** naively learns the vision-force policy with RL.

**Figure 6:**
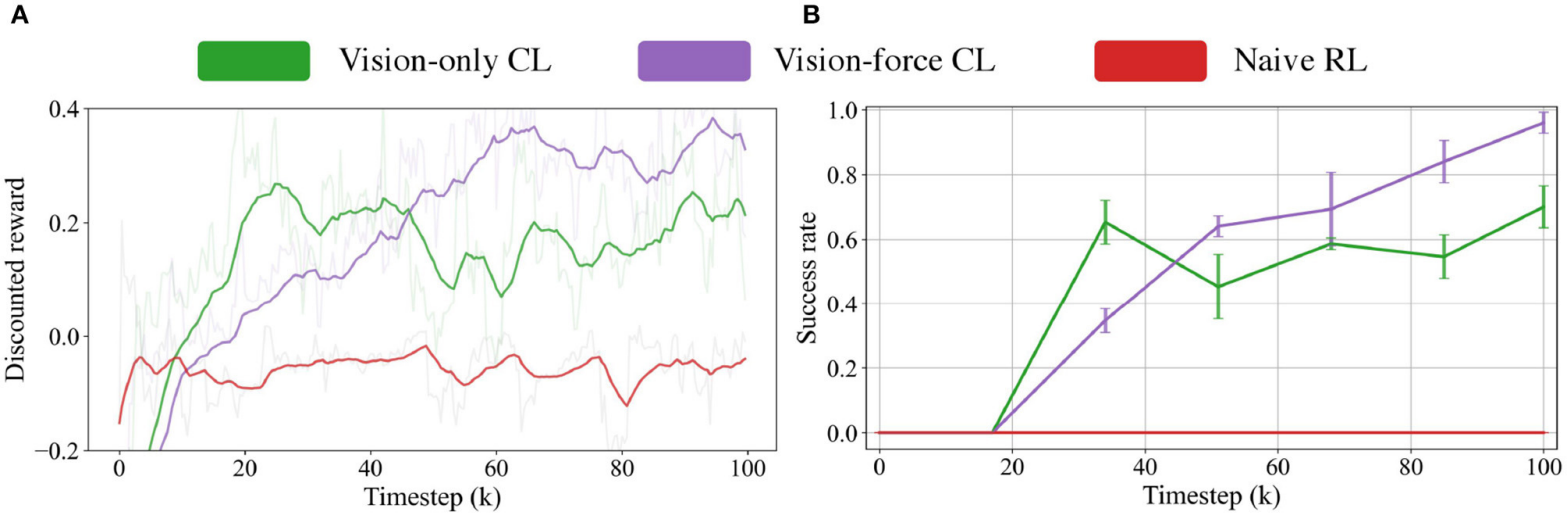
**(A)** Training curves of three models, including the Vision-only CL model, Vision-force CL model, and Naive RL model. **(B)** The insertion success rates at different training stages of three models.

All of the above-mentioned models are trained and tested in simulation. For a fair comparison, all the models except the *Naive RL model* are initialized using a pure visual policy trained with a larger clearance. Figures 6A, B visualize the learning curves during the training and the test results for 250 trials with three random seeds.

#### 5.3.1. Vision-force vs. vision-only

The experiment results indicate the superior performance of the *Vision-force CL model* over the *Vision-only CL model*, manifesting the necessity of vision-force fusion in contact-rich precise manipulation tasks. As demonstrated in Figure 6, comparing the *Vision-force CL model* with the *Vision-only CL model*, the proposed method achieves more than 20% improvement in success rate (70% → 95.2%). Although the ablative *Vision-only CL model* doesn't perform as well as *Vision-force CL model*, it maintains a success rate of 70% which indicates that integrating sensor-based controllers is a solution for contact-rich tasks. Formulating the assembly task as a servoing problem and solving it with curriculum policy learning end-to-end is a good fit for the challenging precise insertion. Nonetheless, the fusion of vision and force perception results in significantly improved outcomes, as the contact-rich insertion task is sensitive to both visual and force signals. Vision perception serves as the main data stream to locate the target, and force perception is a complementary data source when contacts are made and interactions occur.

#### 5.3.2. CL-based model vs. naive RL model

In terms of the results of the CL, the experiment results indicate that the conduct of CL is decisive for multimodal strategy generation in extremely challenging tasks. Comparing the *Vision-force CL model* with the *Naive RL model* in Figure 6, the proposed method could achieve a remarkable success rate of 95.2%. In contrast, the ablative *Naive RL model* couldn't succeed in the task and has 0% success rate. The huge performance gap between the two models comes from the different policy learning formulations. The *Naive RL model* leverages visual and force data to insert the square peg whose clearance is as low as 0.1 mm from scratch. Nevertheless, it's difficult for the agent to coordinate the motions and insert the peg into the hole as a rash motion will cause the slippery of the peg and finally lead to the local optima of the algorithm. Different from the naive RL modeling, the CL-based modeling first learns a visual policy on a larger clearance and is followed by the fusion of force perception on a 0.1 mm clearance task. The curriculum task difficulty organization provides a more effective policy generation approach.

### 5.4. Physical robot experiments

**Figure 7:**
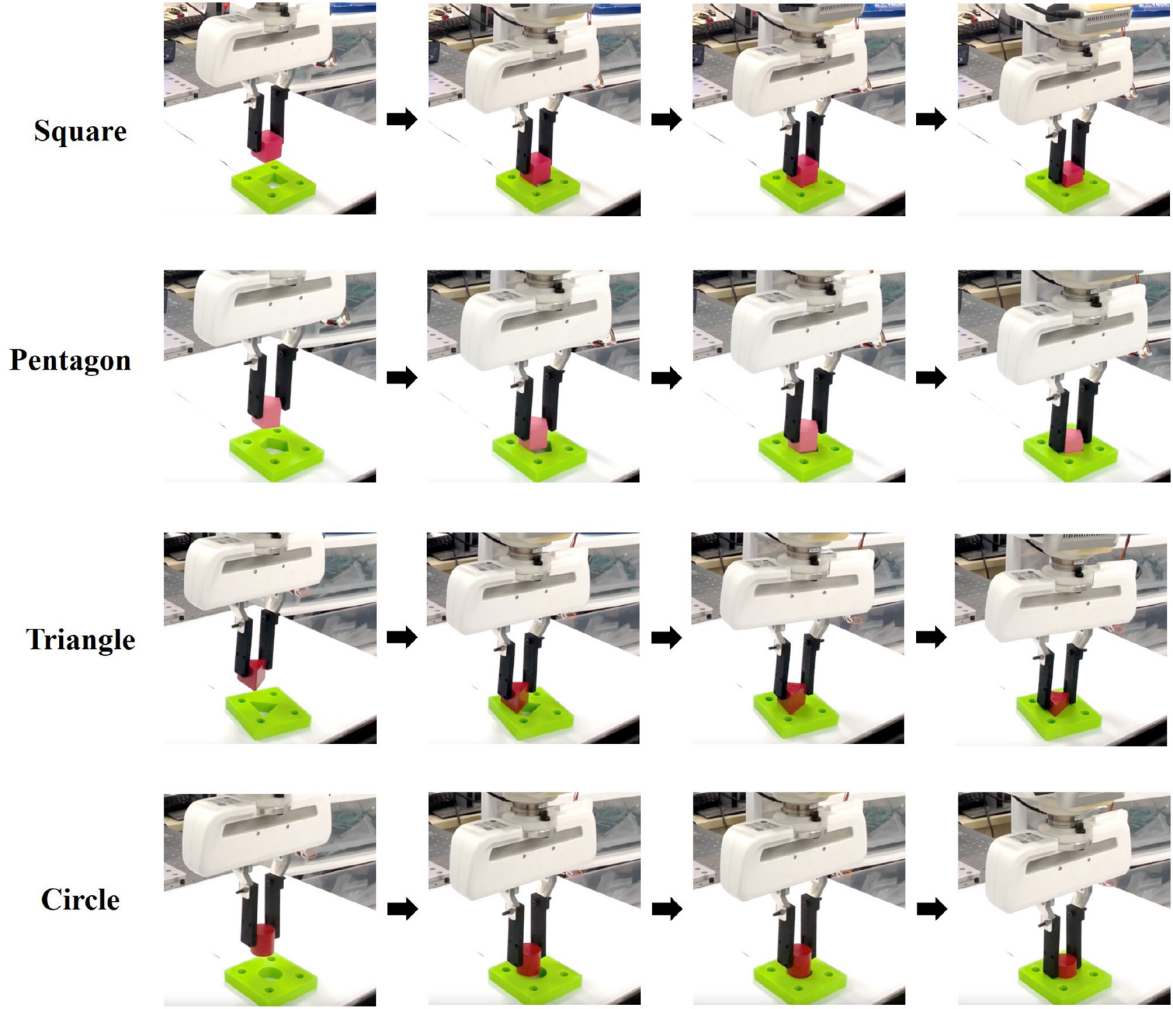
Snapshots of the peg-hole insertion process during the physical robot experiments.

For **RQ4**, we perform direct sim-to-real transfer and generalization tests on the real machine. In the experiment, the robot first grasps the object and then executes the assembly policy to insert the peg into the hole. The insertion hole is rigidly fixed so as not to add extra compliance to the system. Figure 7 shows the four shapes utilized in our experiments, along with snapshots captured during the insertion process. Specifically, the square, pentagonal, triangular, and round peg-hole clearances are 0.37 mm, 0.44 mm, 1 mm, and 0.41 mm, respectively. Table 2 presents the results obtained from the experiments on these four shapes using two models: the *Vision-only CL model* and the *Vision-force CL model*. Experiment results indicate that the simulated assembly system can be transferred to the physical robot. Moreover, the *Vision-force CL model* demonstrates stronger robustness against the ablative *Vision-only CL model*. As shown in Table 2, the *Vision-force CL model* achieves 20% success rate more than the *Vision-only CL model*. Although the *Vision-only CL model* could be transferred to the physical robot, the *Vision-force CL model* even demonstrates better behavior. The performance gap between the two models is consistent with that in the simulated system. Although dynamics in the simulated and physical environment differ, the domain randomization techniques applied to the visual encoder and the compliant motion/force controller to handle uncertain contacts minimize the reality gap. Furthermore, consistent with the situation in simulations, the method could also be generalized to unseen shapes in physical environments.

**Table 2:** Performance on physical assembly task.

**Models Shapes**	**Square**	**Pentagon**	**Triangle**	**Circle**
Vision-only CL	3/10	8/10	3/10	2/10
Vision-force CL	6/10	9/10	5/10	4/10

## 6. Conclusion

This paper proposes a novel vision-force fusion scheme for contact-rich precise assembly tasks. Our approach utilizes a curriculum policy learning mechanism to effectively fuse multi-view visual and force features and implement compliant motions. By effectively fusing visual and force data from perception to control, our method achieves higher precision and better generalization to unseen shapes in the simulated environment. The experiments on the physical environment validate the practicability of our simulated system. Our vision-force system significantly contributes to the advancement of multimodal contact-rich tasks.

## Data availability statement

The raw data supporting the conclusions of this article will be made available by the authors, without undue reservation.

## Author contributions

PJ: Investigation, Methodology, Writing—original draft, Writing—review and editing. YL: Methodology, Writing—review and editing. YS: Writing—review and editing. TL: Funding acquisition, Writing—review and editing. WY: Writing—review and editing.
